# Supplementary material for: Neural and computational underpinnings of biased confidence in human reinforcement learning
Source: Nat Commun. 2023 Oct 28;14:6896. doi: 10.1038/s41467-023-42589-5 (PMC10613217; doi:10.1038/s41467-023-42589-5)
Supplement: Supplementary file 1 — Supplementary Information [file 41467_2023_42589_MOESM1_ESM.pdf]

Supplementary

Table of Contents

Supplementary Results *2*

Computational modelling - results **2**

fMRI – supplementary results **8**

Supplementary tables *10*

Supplementary Table S1. Demographics (a) and descriptive statistical results of behavioral data (b) 10
Supplementary Table S2. Repeated measures ANOVA results reported for learning performance 10
Supplementary Table S3. Estimated coefficients from generalized linear mixed-effect models (GLME) on confidence 11
Supplementary Table S4. Correlation between confidence and RT 12
Supplementary Table S5. Comparison of Ting et al., 2020 and current estimated coefficients from inter-individual robust regressions. 12
Supplementary Table S6. Repeated measures ANOVA results reported for ROI analysis (GLM1 during symbol presentation) 13
Supplementary Table S7. fMRI model-free analysis – GLM1 15
Supplementary Table S8. fMRI model-based analysis – GLM3 16
Supplementary Table S9. fMRI model-based analysis – GLM4 17

Supplementary References *18*

Supplementary Results

Computational modelling - results

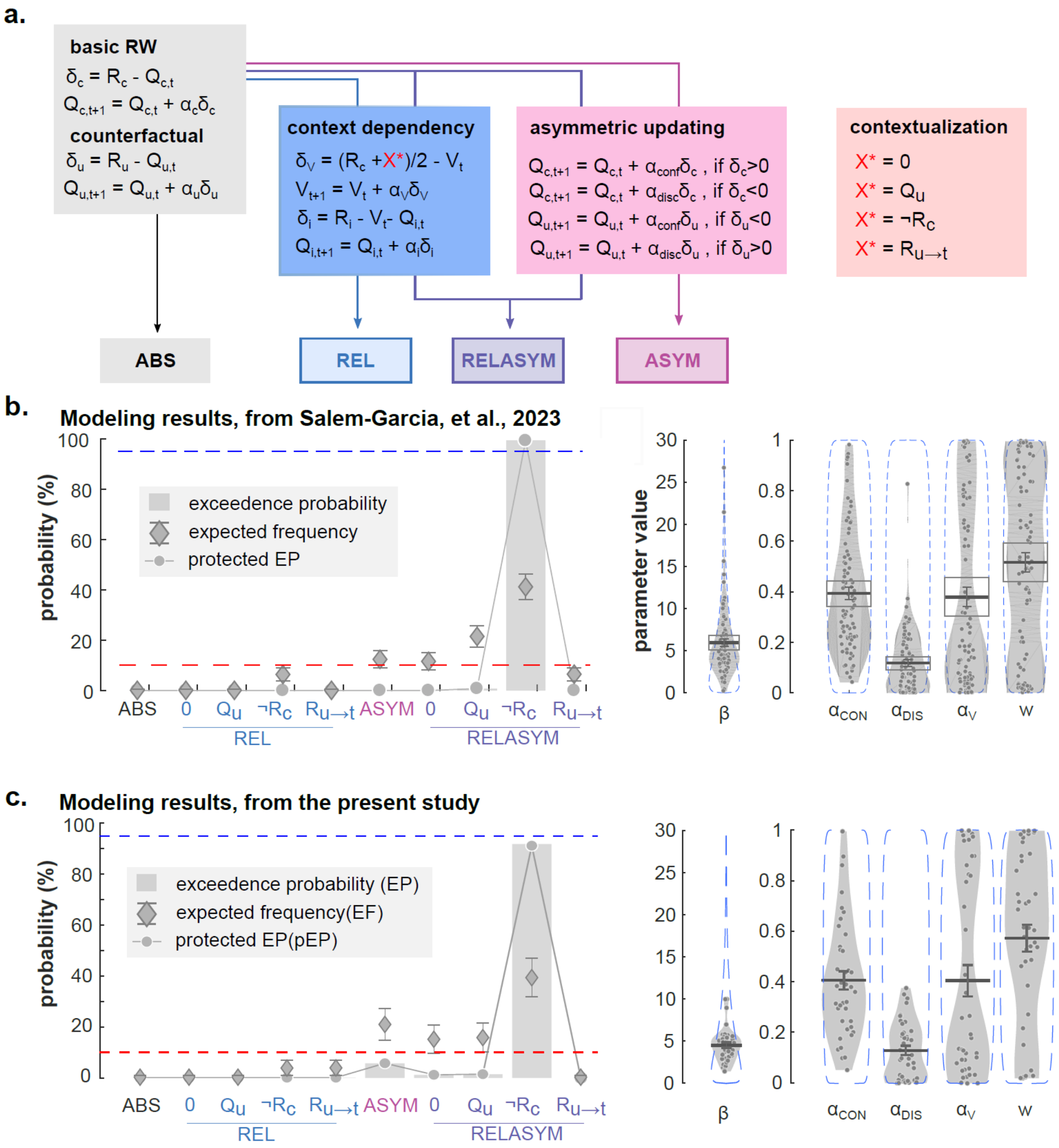

Supplementary Figure S1 | Modeling approach and results. (a) The model architecture and family Bayesian Model Comparison (BMC) procedure. Color panels represent different components of value updating rules. Gray panel: Absolute model (ABS), which consists of basic Rescorla-Wagner (RW) update rule. This rule updates chosen and unchosen option values via outcome directly. Blue panel: Relative model (REL), which consists context-dependent component and updates option values by considering context value. Pink panel: Asymmetric updating model (ASYM), which updates option values based on the valence of prediction error. Purple panel: relative-asymmetric model (RELASYM), which is the combination of relative model and asymmetric updating model. The contextualization panel is used to update unchosen option in the partial information condition. Specifically, X* is determined as the unchosen option outcome (Ru) when the value is available in the complete information condition. When the unchosen option outcome (Ru) is not available in the partial information condition, X* is hypothesized as none (0), expected unchosen value (Qu), paired outcome (¬R) and last seen outcome associated with the option (Ru→t). **(b)** Modeling results from [1] (n = 90 independent participants) and **(C)** from the present study (n = 40 independent participants). **(b-c)** Left panels: Bayesian model comparison. X-

axis represents the models with different hypothesized contextualization values. Y-axis represents the value of three criteria, including exceedance probability (grey histograms), expected frequencies (diamonds) and protected exceedance probability (line & dots) of each model. The red dashed line represents the guessing level for EF and LF. The blue dashed line represents the threshold (95%) for the exceedance probability. **(b-c)** Right panels: Estimated parameter values of the winning model (ASYMREL, X* = with ¬Rc) from [1] **(b)** and from the present study **(c).** Dots represent individual data points. Error bars displayed within the violin plots indicate the sample mean ± SEM. The blue, dotted envelop represent the prior distribution.

$Q_{c/u,t}$: value of the chosen/unchosen option at trial t. $R_{c/u}$: outcome associated to the chosen/unchosen option. $\delta_{c/u}$: prediction error for the chosen/unchosen option. $\alpha_{u/c}$: learning rate for the chosen/unchosen option. $\alpha_{conf/disc}$: learning rate for confirmatory/disconfirmatory information. V_t: context value; $\delta_{V:}$ prediction error for the context value. α_V: learning rate for the context value.

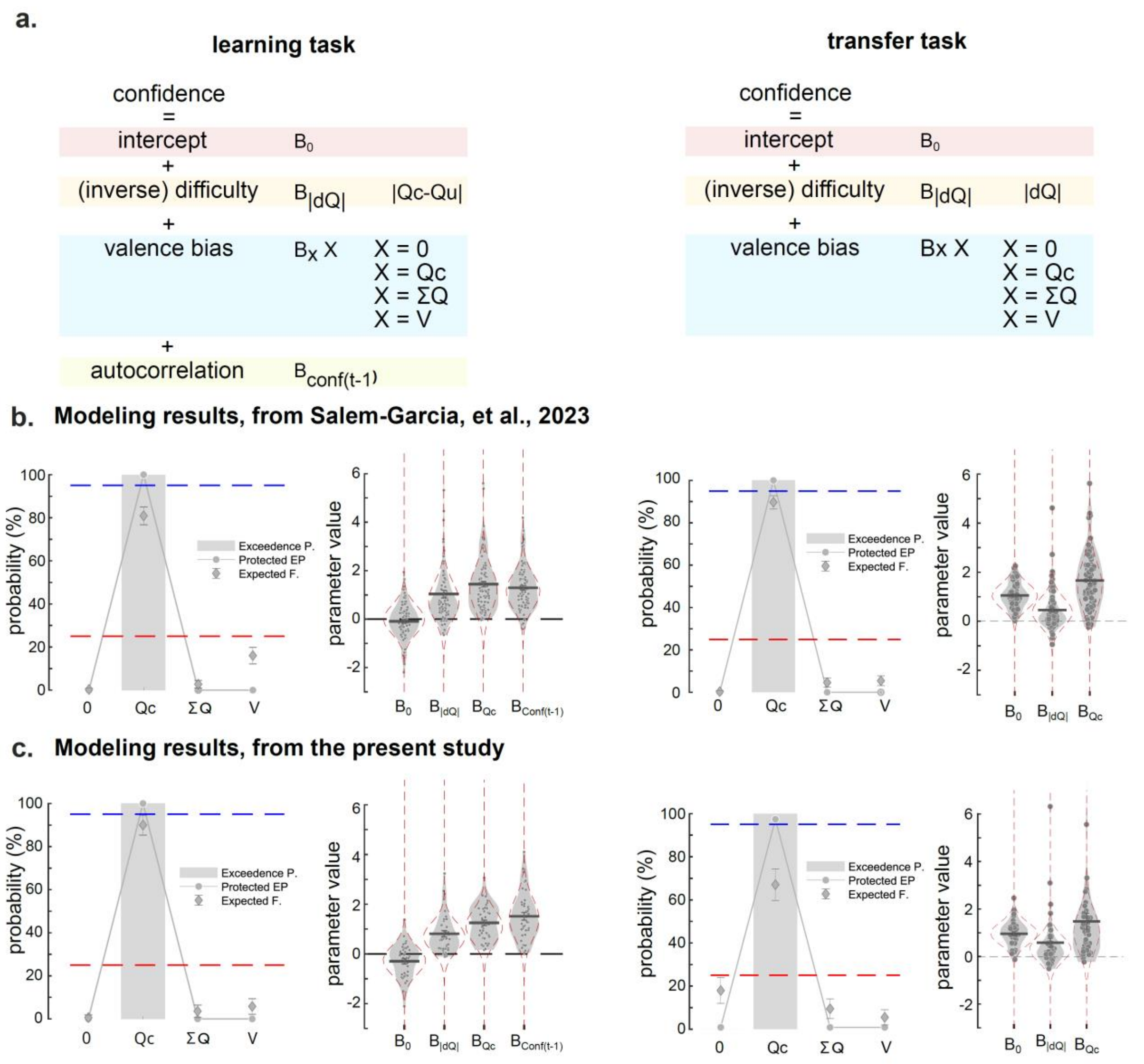

Supplementary Figure S2 | Modeling confidence in the learning task and transfer task. (a) Confidence models are linear regression models. Left panel: Each model in the learning task consists of an intercept and three slopes, which accounts for difficulty (i.e., absolute value difference of present options), source of valence bias, and autocorrelation (i.e., confidence ratings in the previous trial). Right panel: Each model in the transfer task consists of an intercept and two slopes, which accounts for difficulty, and source of valence bias. In different models, the source of valence bias (X) is hypothesized as none, or summed value of present options, or context value, or expected chosen option value. These values are estimated by the winning RL model. **(b)** Modeling results from [1] and **(c)** from the present study. **(b-c)** Left panels of the learning task and the transfer task: Bayesian model comparison. X-axis represents the models with different hypothesized source of confidence bias. Y-axis represents the value of three criteria, including exceedance probability (grey histograms), expected frequencies (diamonds) and protected exceedance probability (line & dots) of each model. The red dashed line represents the guessing level for EF and LF. The blue dashed line represents the threshold (95%) for the exceedance probability. **(b-c)** Right panels of the learning task and the transfer task: Estimated regression coefficients of the winning model (with valence bias =Qc) from [1] **(b)** and from the present study **(c).** Dots represent individual data points (n = 40 independent participants). Error bars displayed within the violin plots indicate the sample mean ± SEM. The red, dotted envelop represent the prior distribution.

Qc: chosen option value; ΣQ: summed present option values; V: context value; β: inverse temperature; $\alpha_{CON/DIS}$: learning rate for confirmatory/disconfirmatory information; α_V: learning rate for the context value; w: partial contextualization weight.

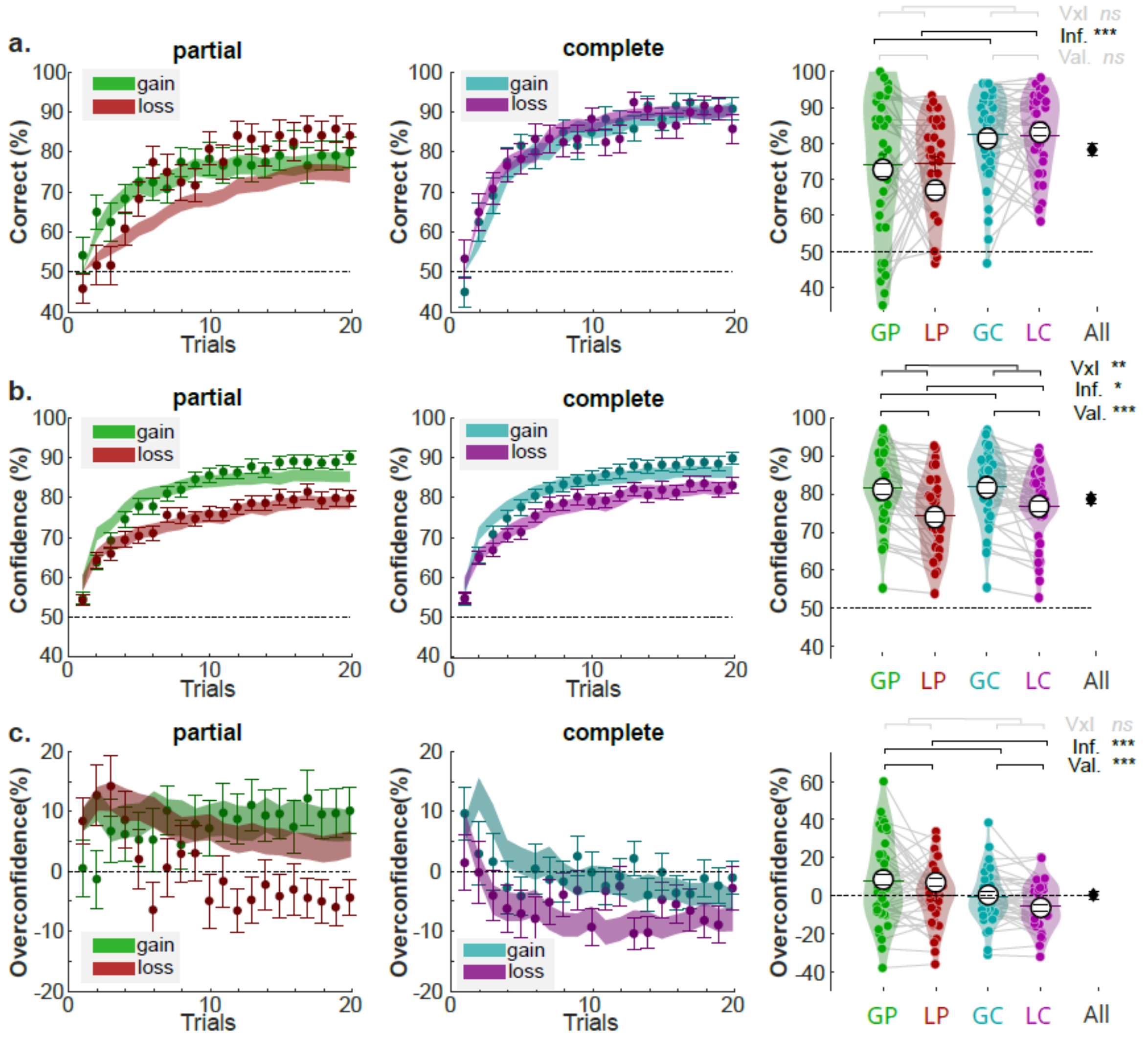

Supplementary Figure S3 | Model predictions and real data in the learning task (a-c). Left and middle panels are trial-by-trial **(a)** percentage of correct responses, **(b)** Confidence rating, and **(c)** overconfidence in the partial information (left panels) and complete information condition (middle panels). Filled colored areas represent mean±SEM. Shaded areas represent the model predictions. **(a-c)** Right panels are average **(a)** percentage of correct responses, **(b)** Confidence rating, and **(c)** overconfidence across conditions at the individual level (dots; n = 40 independent participants) and group-level (horizontal bars). The black error bars indicate the overall performance over conditions. White circles and error bars displayed within the violin plots indicate mean ± SEM of the model predictions.

G75 and G25: options associated with 75% and 25% probability of winning the big gain (1€), respectively; L75 and L25: options associated with 75% and 25% probability of losing the big loss (-1€), respectively.

~: .05<*P*<.1; *: .01<*P*<.05; **: .001<*P*<.01; ***: *P*<.001

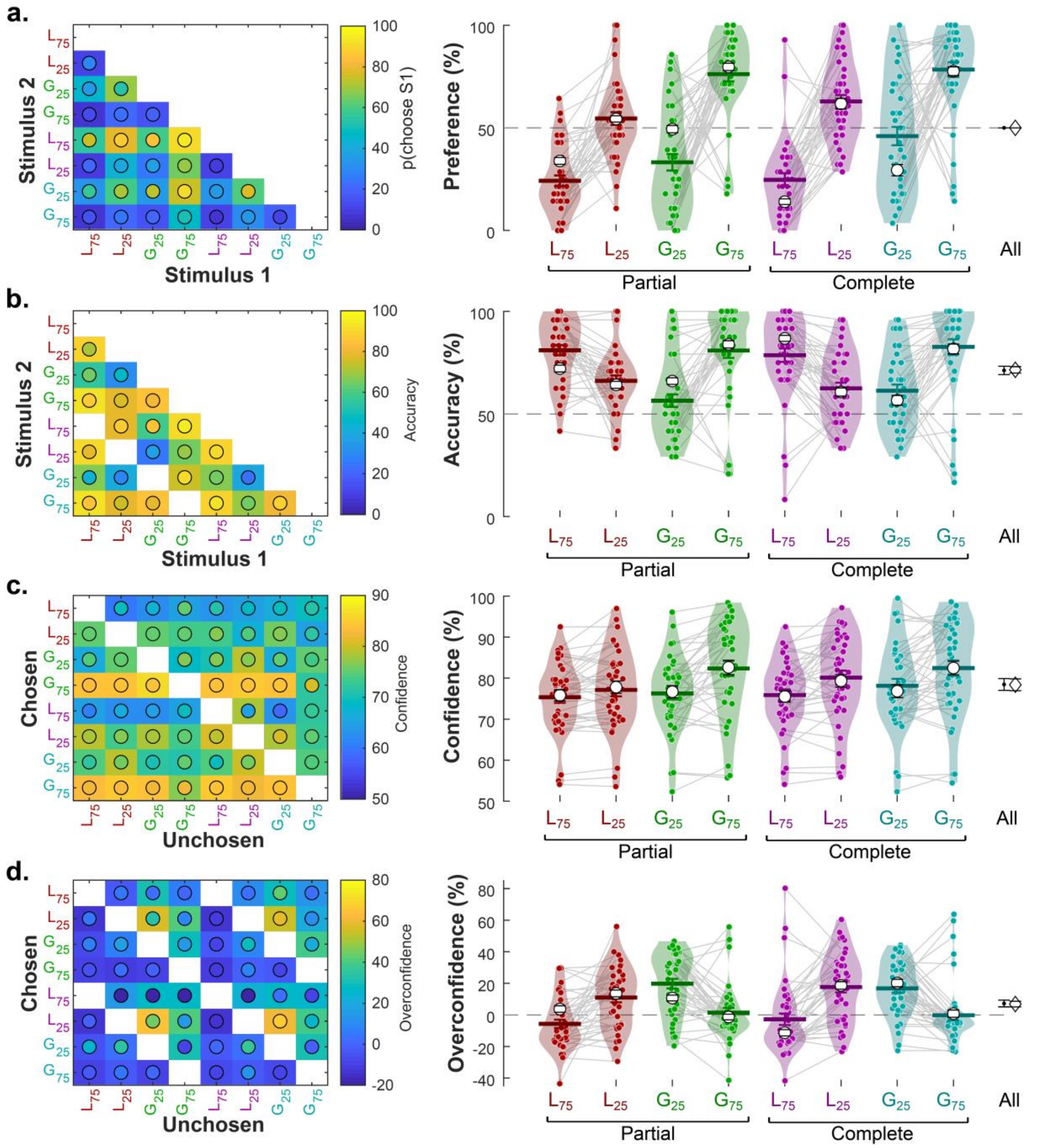

Supplementary Figure S4 | Model predictions and real data in the transfer task. (a-d) Left: Color-coded preference **(a)**, accuracy **(b)**, confidence **(c)** and overconfidence **(d)** for each pair of option presented in the transfer task. Lego plot pictures color-coded confidence for each pair of option presented in the transfer task. Background (square) color represent behavior, while overlaid colored circles represent model predictions. Right: Individual averaged confidence for each option (i.e., averaged over every choice including the option). Connected dots represent individual data points in the within-subject design (n = 40 independent participants). Error bars displayed within the violin plots indicate the sample mean ± sem. Rightmost black diamond and error bar represents the average over all conditions (mean ± SEM). White circles and error bars represent mean ± SEM of the model predictions.

G75 and G25: options associated with 75% and 25% probability of winning the big gain, respectively; L75 and L25: options associated with 75% and 25% probability of losing the big loss, respectively.

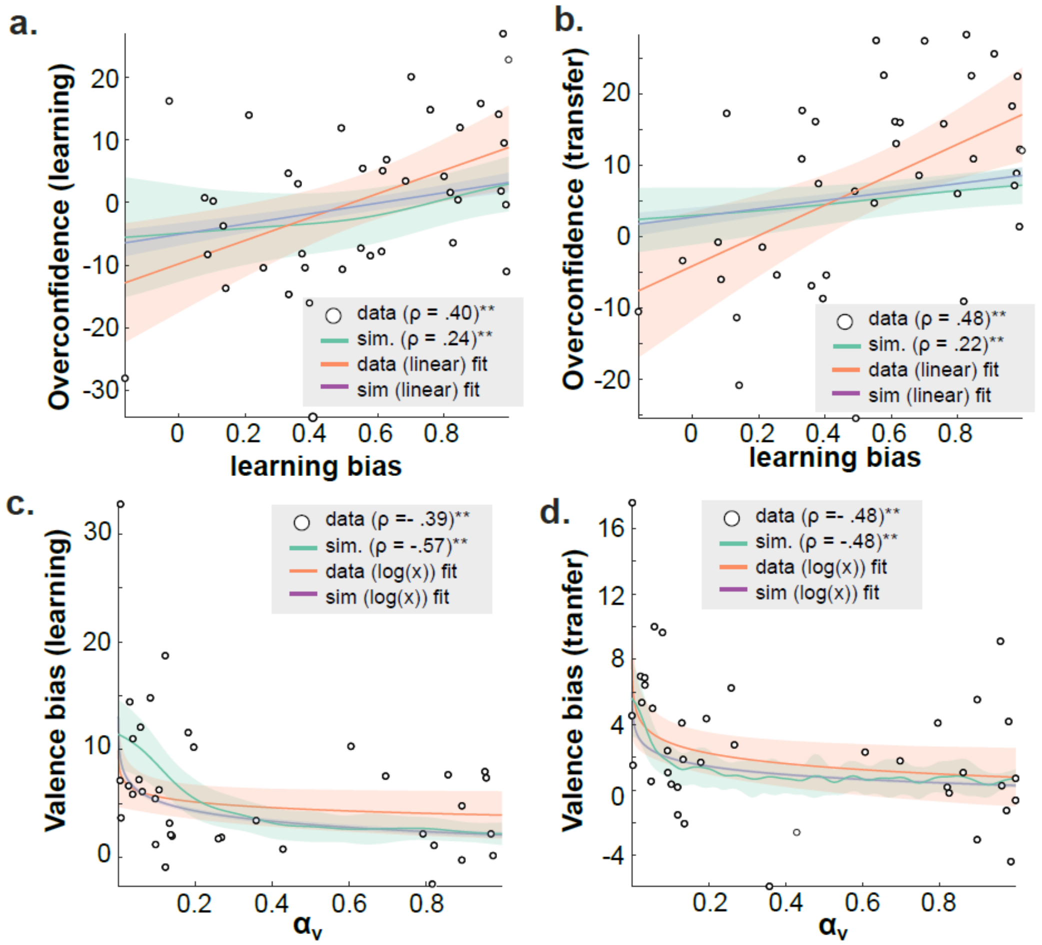

Supplementary Figure S5 | Correlations between learning model parameters and confidence biases. The scatter-plots depict correlations estimated in both the learning **(a, c)** and the transfer **(b, d)** tasks, between learning bias ((α_{CON} - α_{DIS})/(α_{CON} + α_{DIS})) and overconfidence **(a, b)** and between contextual learning rate (α_V) and valence-induced confidence bias **(c, f)**. Dots represent individual estimates (n = 40 independent participants). Color lines and shaded areas represent non-parametric regression estimate ± CI 95%, obtained from simulations (see Methods).

~: .05<*P*<.1; *: .01<*P*<.05; **: .001<*P*<.01; ***: *P*<.001

fMRI – supplementary results

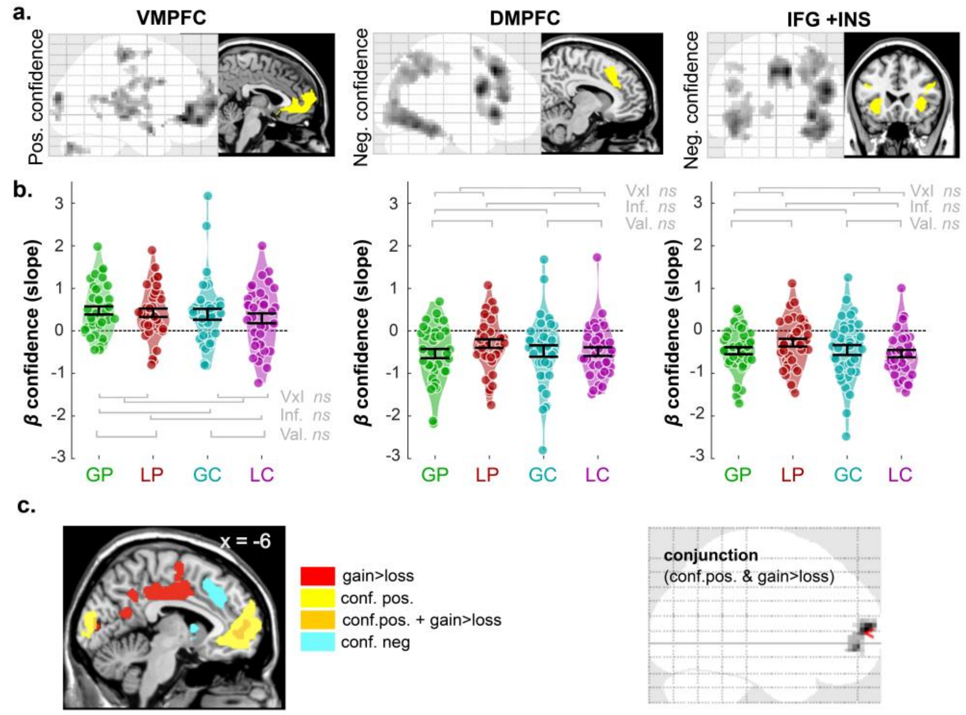

Supplementary Figure S6 | confidence encoding in the human brain. (a) Results of GLM1 whole-brain analysis, reproduced from **Figure 3a**, for convenience. Brain areas positively (left panels) and negatively (middle and right panels) correlate with confidence rating during the symbol presentation phase. Significant voxels are displayed on the glass brains in a gray-to-black gradient manner (one-sided tests; $p_{uncorrect}< .001$, cluster size >47). The yellow areas in the anatomical brain are ROIs (vmPFC, dmPFC, and IFG+INS), which are used in the following ROI analyses. **(b)** Violin plots represent the sample distribution of fMRI regression coefficients of parametric confidence signals for the different contexts (represented by different colors), in the ROI depicted in **(a)**. Dots correspond to individual regression coefficients (n = 38 independent participants). Error bars represent sample mean ± SEM. GP: gain/partial; LP: loss/partial; GC: gain/complete; LC: loss/complete. Two-way repeated-measures ANOVAs detected no effect of our experimental manipulation on parametric confidence signals in any of our ROIs. **(c)** Left: superposition of GLM1 significant clusters, for three contrasts: [gain>loss], red; [positive effect of parametric confidence], yellow; [negative effect of parametric confidence]. Only the first two overlapped (orange). Significant clusters defined as: one-sided tests; voxel-wise $P_{uncorrected} < .001$; cluster-wise $P_{FWE} < .05$. Right: formal whole brain analysis of a conjunction between [positive effect of parametric confidence] and [gain vs loss], (one-sided tests; voxel-wise $P_{uncorrected} < .001$; cluster-wise $P_{FWE} < .05$).
The brain depicted in the figure is based on a template from the software MRIcron. Chris Rorden's MRIcron, all rights reserved. https://people.cas.sc.edu/rorden/mricron/install.html”

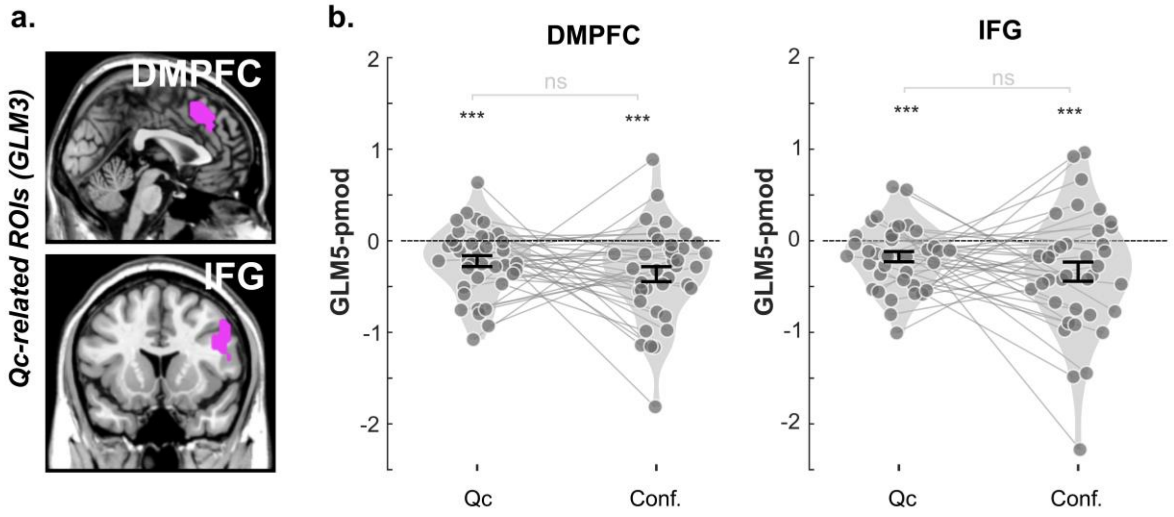

Supplementary Figure S7 | Activations in the negative networks cannot be better explained by confidence. (a) ROI analysis with Qc-related ROIs identified in the present study (purple areas). **(b)** The regression coefficients corresponding to Qc and confidence in GLM5 were summarized at the individual level (dots; n = 38 independent participants) for ROI of DMPFC and IFG+INS, separately. Error bar represents SEM. Two-sided paired t-test detected no significant difference in the encoding of confidence versus Qc in the regions of the negative network (DMPFC: $t_{37} = 1.02$, P = .2352; IFG: $t_{37} = 1.19$, P = .2390).

Qc: parametric modulator of chosen option; Conf.: parametric modulator of confidence ratings

~: .05<*P*<.1; *: .01<*P*<.05; **: .001<*P*<.01; ***: *P*<.001

The brain depicted in the figure is based on a template from the software MRIcron. Chris Rorden's MRIcron, all rights reserved. https://people.cas.sc.edu/rorden/mricron/install.html”

Supplementary tables

a.

**Gender** M/F	**Age** mean ± STD	**Performance (%)** mean ± SEM	**Confidence (%)** mean ± SEM	**RT (ms)** mean ± SEM	**Conf. RT** mean ± SEM
17/23	22.69±4.44	78.31 ± 1.59	78.71 ± 1.33	715.76 ± 33.76	1948±68.13

b.

	Gain Partial	Loss Partial	Gain Complete	Loss Complete
**Performance (%)** mean ± SEM	74.12 ±0.48	74.42 ± 0.32	82.42 ± 0.31	82.29 ± 0.27
**Confidence (%)** mean ± SEM	81.63 ± 0.23	74.38 ± 0.24	81.92 ± 0.23	76.89 ± 0.23
**Confidence bias** mean ± SEM	7.51 ± 3.50	-0.04 ± 2.45	-0.49 ± 2.20	-5.41 ± 3.50
**RT (ms)** mean ± SEM	693.02 ± 34.02	745.91 ± 38.02	698.31 ± 34.34	725.79 ± 38.20
**Conf. RT (ms)** mean ± SEM	1960±66.58	1909±68.38	1980±74.42	1943±74.04

Supplementary Table S1. Demographics (a) and descriptive statistical results of behavioral data (b)

M: Male; F: Female; STD: standard deviation; SEM: standard error of the mean

	Valence	Information	Valence × Information
**Performance** F(1,39) [η^2] (*p*-val.)	0.00 [0.00] (.9666)	22.05 [0.07] (<.0010)***	0.01 [0.00] (.9056)
**Confidence** F(1,39) [η^2] (*p*-val.)	36.56 [0.10] (<.0010)***	6.76 [0.00] (.0131)*	9.62 [0.00] (.0036)**
**Confidence bias** F(1,39) [η^2] (*p*-val.)	12.28 [0.03] (.0012)**	14.42 [0.04] (<.0010)***	0.58 [0.00] (.4506)
**RT** F(1,39) [η^2] (*p*-val.)	4.77 [0.00] (.0350)*	0.31 [0.00] (.5782)	0.97 [0.00] (.3318)
**Conf. RT** F(1,39) [η^2] (*p*-val.)	3.42 [0.00] (.0722)~	1.62 [0.00] (.2112)	0.09 [0.00] (.7691)

Supplementary Table S2. Repeated measures ANOVA results reported for learning performance

Valence: gain/loss; Information: partial/complete. ~ $p<.10$; * $p<.05$; ** $p<.01$; *** $p<.001$, all two-sided.

		GLME1	GLME2	GLME3	GLME4
**Valence**	β±SE	4.95±1.06	2.55±0.55	5.57±1.19	2.96±0.63
	t-val	4.66	4.61	4.66	4.67
	(*p*-val)	(<.001)***	(<.001)***	(<.001)***	(<.001)***
**Information**	β±SE	-2.44±0.67	-1.16±0.36	-2.27±0.75	-1.27±0.40
	t-val	-3.60	-3.21	-2.99	-3.14
	(*p*-val)	(<.001)***	(.001)**	(.002)**	(.001)**
**Valence × Information**	β±SE	2.13±0.70	1.03±0.43	1.83±0.83	1.10±0.43
	t-val	3.02	2.38	2.19	2.51
	(*p*-val)	(<.001)***	(.017)*	(.028)*	(.011)**
**RT**	β±SE	-0.00±0.00	-0.00±0.00	-0.00±0.00	-0.00±0.00
	t-val	-13.94	-11.65	-13.22	-11.62
	(*p*-val)	(<.001)***	(<.001)***	(<.001)***	(<.001)***
**Session**	β±SE		0.52±0.24	0.61±0.21	0.42±0.20
	t-val		2.17	2.18	2.02
	(*p*-val)		(.029)*	(.002)**	(.042)*
**Previous confidence (regardless of conditions)**	β±SE			0.42±0.00	0.12±0.00
	t-val			50.32	14.16
	(*p*-val)			(<.001)***	(<.001)***
**Previous confidence (Within condition)**	β±SE		0.55±0.00		0.49±0.00
	t-val		72.31		57.40
	(*p*-val)		(<.001)***		(<.001)***
**R^2**		0.3260	0.6156	0.4970	**0.6238**

Supplementary Table S3. Estimated coefficients from generalized linear mixed-effect models (GLME) on confidence

β: estimated regression coefficients for fixed effects. SE: estimated standard error of the regression coefficients.
~ p <.1; * p <.05; ** p <.01; *** p <.001, all two-sided.

	Gain partial	Loss partial	Gain complete	Loss complete	Overall
mean ± SEM	-0.18 ± 0.03	-0.21 ± 0.03	-0.18 ± 0.02	-0.20 ± 0.03	-0.19 ± 0.02
t(39)	-5.56	-6.13	-6.63	-5.86	-8.21
(*p*-val)	(<.001)***	(<.001)***	(<.001)***	(<.001)***	(<.001)***

Supplementary Table S4. Correlation between confidence and RT

The correlation between confidence and reaction time was performed at the session level using Pearson's R, then averaged at the individual level. Reported statistics correspond to a random-effects analysis (one sample t-test) performed at the population level.

SEM: standard error of the mean. t: Student t-value.

~ *p*<.10; * *p* <.05; ** *p* <.01; *** *p* <.001, all two-sided.

		**Ting, et al 2020 [2]**						**current data**
		Exp. 1	Exp. 2	Exp. 3	Exp. 4	Exp. 5	Exp. 6	
**Intercept**	β ± SE	8.02 ± 2.15	2.94 ± 1.29	2.16 ± 0.95	3.54 ± 1.37	6.76 ± 1.81	3.59 ± 1.95	5.02 ± 0.84
	t-val	3.72	2.27	2.27	2.58	3.73	2.41	5.97
	(*p*-val)	(.002)**	(.037)*	(.038)*	(.020)*	(.002)**	(.028)*	(<.001)***
**Slope**	β ± SE	-0.00±0.01	-0.03 ± 0.01	-0.02 ± 0.04	-0.06 ± 0.06	0.17 ± 0.18	-0.10 ± 0.03	-0.01± 0.01
	t-val	-0.27	-3.55	-0.46	-0.97	0.81	-3.35	-0.97
	(*p*-val)	(.793)	(.003)**	(.662)	(.35)	(.368)	(.004)**	(.339)

Supplementary Table S5. Comparison of Ting et al., 2020 [2] and current estimated coefficients from inter-individual robust regressions.

For each individual, we estimated the net effect of valence on RT and confidence, by computing the averaged difference of these behavioral measures in the gain versus loss contexts. The fMRI experiment with larger sample size (n=40; selected by a orange frame) revealed the similar results as we found from out previous result : Experiment 1-6 in [2].

β: estimated regression coefficient. SE: estimated standard error of the regression coefficient.

~ *p*<.10; * *p* <.05; ** *p* <.01; *** *p* <.001, all two-sided.

cue-evoked		Valence	Information	Valence × Information
**VMPFC**	F(1,37) [η^2] (*p*-val.)	8.99 [0.020] (.0048)**	0.34 [0.000] (.5602)	3.99 [0.008] (.0532)~
**DMPFC**	F(1,37) [η^2] (*p*-val.)	3.05 [0.007] (.0893)~	0.30 [0.000] (.5849)	0.22 [0.000] (.6449)
**IFG+INS**	F(1,37) [η^2] (*p*-val.)	0.13 [0.000] (.7119)	0.03 [0.00] (.8721)	0.04 [0.00] (.8421)
Pmod: confidence		Valence	Information	Valence × Information
**VMPFC**	F(1,37) [η^2] (*p*-val.)	0.36 [0.003] (.5502)	1.56 [0.006] (.2194)	0.03 [0.000] (.8429)
**DMPFC**	F(1,37) [η^2] (*p*-val.)	1.23 [0.006] (.2752)	1.13 [0.002] (.2938)	0.97 [0.008] (.3317)
**IFG+INS**	F(1,37) [η^2] (*p*-val.)	0.34 [0.002] (.5629)	4.26 [0.010] (.0459)	2.41 [0.013] (.1287)

Supplementary Table S6. Repeated measures ANOVA results reported for ROI analysis (GLM1 during symbol presentation)

~ $p<.10$; * $p<.05$; ** $p<.01$; *** $p<.001$ with Bonferroni corrected for 3 comparisons.

Parametric effect	Regions	MNI coordinates			Cluster size	T
		x	y	z		
Confidence (Positive effect)	pregenual anterior cingulate cortex (pgACC)	3	35	-7	697	6.65
		0	41	2		6.11
		-9	50	-7		6.00
	Calcarine sulcus	-6	-91	14	79	5.42
		-3	-94	-4		4.23
		-12	-91	5		4.18
	Precentral gyrus	-30	-25	59	139	5.37
		-18	-31	62		3.88
		-45	-25	56		3.58
	Middle temporal gyrus	-57	-25	-4	65	5.21
		-48	-31	2		4.22
	Middle temporal gyrus	60	2	-19	397	5.18
		66	-19	2		4.94
		69	-31	8		4.68
	Cerebellum	-27	-79	-34	68	5.07
		-21	-85	-43		3.90
	Middle frontal gyrus	24	11	32	130	4.87
		27	-1	26		4.57
		21	-19	29		4.46
	Cerebellum	27	-79	-34	86	4.83
		42	-70	-40		4.11
		45	-61	-40		3.72
	Superior temporal gyrus	-51	-34	20	73	4.60
		-63	-46	14		4.54
		-60	-55	17		4.09
	Precentral gyrus	21	-31	59	58	4.51
		30	-28	62		4.22
	Frontal gyrus	15	-19	62	61	4.50
		6	-1	62		4.48
		21	-19	56		4.05
Confidence (Negative effect)	Dorsal medial prefrontal cortex (DMPFC)	9	23	41	324	8.97
		-3	26	41		7.83
		0	23	53		5.15
	Inferior frontal gyrus (IFG)	48	8	23	405	8.41
		45	32	14		6.62
		45	29	23		5.87
	Insula (INS)	33	23	-4	110	7.94
		48	14	2		3.77
	Fusiform gyrus	45	-58	-16	1232	7.44
		30	-40	-19		6.71
		42	-43	-22		6.49
	Fusiform gyrus	-42	-70	-10	522	6.76
		-45	-55	-13		6.35
		-42	-46	-19		6.29
	Precentral gyrus	33	5	62	72	5.27
		36	17	59		3.47
	Inferior frontal gyrus	-48	35	23	67	5.25

	(lIFG)	-42	26	23		4.69
	Insula (lINS)	-30	26	-7	132	5.21
	Precentral gyrus	-42	5	29	86	4.58
		-48	11	26		4.24
	Caudate	-9	11	-1	61	4.25
	Intraparietal sulcus	-39	-46	44	56	4.12
		-48	-40	44		3.83
Interaction (LP>GP – GP>LP)	Paracentral lobe	0	-25	74	59	4.34
		3	-25	65		4.16
		-9	-22	71		3.94

**Cue-evoked**	**Regions**	**MNI coordinates**			**Cluster size**	**T**
		**x**	**y**	**z**		
Gain>Loss	Cingulate gyrus	-6	-10	38	1108	5.38
		-9	-1	44		5.12
		-33	-10	41		5.11
	Middle temporal gyrus	42	-64	8	252	5.25
		51	-52	11		4.92
		63	-46	11		4.88
	Inferior occipital gyrus	27	-70	-10	296	5.09
		24	-82	14		4.55
		15	-88	11		4.54
	Middle temporal gyrus	-54	-64	14	58	4.96
		-45	-64	23		4.36
	Superior temporal gyrus	51	-4	-13	47	4.84
		60	2	-13		4.15
		63	-7	-10		3.88
	Superior occipital gyrus	-15	-88	11	82	4.31
		-6	-88	8		4.10
		-18	-91	2		4.04
	Anterior cingulate cortex	-9	50	2	80	4.28
		18	53	2		4.03
		6	50	-1		3.95

**Conjunction**	**Regions**	**MNI coordinates**			**Cluster size**	**T**
		**x**	**y**	**z**		
Cue-evoked: G>L + Positive confidence effect	Superior frontal gyrus	-6	59	8	105	4.29
	Orbital frontal cortex	-6	53	-4		3.94
	Orbital frontal cortex	6	53	-4		3.54

Supplementary Table S7. fMRI model-free analysis – GLM1

Note: Whole-brain cluster-defining height threshold at uncorrected p <.001, k = 47. With the same threshold, no brain region survives in the contrast of [gain>loss], [complete>partial] nor the contrast of interaction. The regions highlighted with red are ROIs used in the main paper.

MNI: Montreal Neurological Institute; Qc: chosen option value; Qu: unchosen option value;

Parametric effect	Regions	MNI coordinates			Cluster size	T
		x	y	z		
Qc (***Positive effect***)	VMPFC	6	38	-4	107	4.25
		-3	41	-1		4.2
		9	47	-7		4
Qc ***(Negative effect)***	DMPFC	0	26	44	253	6.62
		-3	35	32		4.06
	Fusiform gyrus	45	-58	-10	262	5.9
		42	-52	-16		5.86
		36	-40	-25		4.34
	Occipital gyrus	-45	-58	-13	361	5.44
		-42	-70	-10		5.39
		-36	-82	-10		5.3
	IFG	45	14	29	262	5.12
		42	5	26		4.75
		48	32	20		4.5
	Thalamus	9	-13	8	64	4.9
		-6	-16	11		3.53
	Striatum	21	8	-7	64	4.68
		15	2	-19		4.59
		18	17	-4		4.01
	Superior parietal lobe	27	-64	47	276	4.54
		39	-49	56		4.49
		24	-58	35		4.46
Qu (***Positive effect***)	n.s.					
Qu (***Negative effect***)	Occipital gyrus	24	-85	17	119	4.67
		12	-94	14		4.5
		15	-88	5		4.17
Context value: V (***Positive effect***)	Lingual gyrus	24	-64	-7	67	4.65
		27	-55	-10		4.64
		24	-55	-19		3.82
	Calcarine	-27	-61	11	71	4.19
		-30	-79	11		4.1
		-24	-91	17		3.98
	Occipital gyrus	39	-76	14	66	3.98
		27	-82	14		3.84
		18	-85	14		3.8
Context value: V (***Negative effect***)	n.s.					

Supplementary Table S8. fMRI model-based analysis – GLM3
Note: Whole-brain cluster-defining height threshold at uncorrected $p < .001$, $k = 47$. The regions highlighted with red are ROIs used in the main paper.
MNI: Montreal Neurological Institute

Parametric effect	Regions	MNI coordinates			Cluster size	T
		x	y	z		
Qc (***Positive effect***)	PCC	-9	-13	41	1022	5.97
		-39	-34	23		5.29
		30	20	17		5.19
	Cerebelum	6	-70	-13	574	5.66
		9	-79	-1		5.23
		21	-85	5		5.03
	Occipital gyrus	-15	-88	5	86	5.31
		-6	-88	8		4.49
	Fusiform	-30	-34	-19	60	4.83
		-36	-22	-16		4.61
		-42	-25	-7		4.22
	VMPFC	-3	53	2	105	4.16
		0	65	11		4.08
		9	50	8		3.83
Qc (***Negative effect***)	n.s.					
Difference: |Qc-Qu| (Positive effect)	n.s.					
Difference: |Qc-Qu| (Negative effect)	Inferior temporal gyrus	48	-52	-13	71	5.18
		42	-58	-13		4.97
		39	-40	-16		3.83
	Occipital gyrus	-36	-88	-7	66	4.61
		-42	-61	-13		4.49
		-39	-73	-10		4.11
Confidence$_{t-1}$ (Positive effect)	Insula	42	-19	5	83	4.99
		36	-22	17		4.48
		51	-10	5		4.13
Confidence$_{t-1}$ (Negative effect)	Cerebelum	36	-49	-28	15452	9.08
		36	-61	-13		8.75
		24	-64	-19		8.4
	Cerebelum	-9	-31	-34	94	4.94
		3	-37	-34		4.8
		-9	-31	-25		4.54
	IFG	-45	44	8	71	4.73
		-48	38	14		4.5

Supplementary Table S9. fMRI model-based analysis – GLM4

Note: Whole-brain cluster-defining height threshold at uncorrected p <.001, k = 47.

MNI: Montreal Neurological Institute

Supplementary References

1. Salem-Garcia, N., Palminteri, S. & Lebreton, M. Linking confidence biases to reinforcement-learning processes. *Psychological Review* **130**, 1017–1043 (2023).
2. Ting, C.-C., Palminteri, S., Engelmann, J. B. & Lebreton, M. Robust valence-induced biases on motor response and confidence in human reinforcement learning. *Cogn Affect Behav Neurosci* (2020) doi:10.3758/s13415-020-00826-0.
