# Supplementary material for: Neural and computational underpinnings of biased confidence in human reinforcement learning
Source: Nat Commun. 2023 Oct 28;14:6896. doi: 10.1038/s41467-023-42589-5 (PMC10613217; doi:10.1038/s41467-023-42589-5)
Supplement: Supplementary file 2 — Reporting Summary [file 41467_2023_42589_MOESM2_ESM.pdf]

nature portfolio

Corresponding author(s): Maël Lebreton, Jan B Engelmann, Chih-Chung Ting

Last updated by author(s): Sep 11, 2023

Reporting Summary

Nature Portfolio wishes to improve the reproducibility of the work that we publish. This form provides structure for consistency and transparency in reporting. For further information on Nature Portfolio policies, see our Editorial Policies and the Editorial Policy Checklist.

Statistics

For all statistical analyses, confirm that the following items are present in the figure legend, table legend, main text, or Methods section.

n/a	Confirmed	
☐	☒	The exact sample size (*n*) for each experimental group/condition, given as a discrete number and unit of measurement
☐	☒	A statement on whether measurements were taken from distinct samples or whether the same sample was measured repeatedly
☐	☒	The statistical test(s) used AND whether they are one- or two-sided<br>*Only common tests should be described solely by name; describe more complex techniques in the Methods section.*
☐	☒	A description of all covariates tested
☐	☒	A description of any assumptions or corrections, such as tests of normality and adjustment for multiple comparisons
☐	☒	A full description of the statistical parameters including central tendency (e.g. means) or other basic estimates (e.g. regression coefficient) AND variation (e.g. standard deviation) or associated estimates of uncertainty (e.g. confidence intervals)
☐	☒	For null hypothesis testing, the test statistic (e.g. *F*, *t*, *r*) with confidence intervals, effect sizes, degrees of freedom and *P* value noted<br>*Give P values as exact values whenever suitable.*
☐	☒	For Bayesian analysis, information on the choice of priors and Markov chain Monte Carlo settings
☐	☒	For hierarchical and complex designs, identification of the appropriate level for tests and full reporting of outcomes
☐	☒	Estimates of effect sizes (e.g. Cohen's *d*, Pearson's *r*), indicating how they were calculated

Our web collection on statistics for biologists contains articles on many of the points above.

Software and code

Policy information about availability of computer code

Data collection	Matlab 2015a, COGENT toolbox (Cogent2000v1.33)
Data analysis	Matlab 2021a, SPM12, SPM's toolbox: MACS v1.3;<br>All custom codes necessary to reproduce our analyses are available, without restriction at https://osf.io/s92tj/

For manuscripts utilizing custom algorithms or software that are central to the research but not yet described in published literature, software must be made available to editors and reviewers. We strongly encourage code deposition in a community repository (e.g. GitHub). See the Nature Portfolio guidelines for submitting code & software for further information.

Data

Policy information about availability of data

All manuscripts must include a data availability statement. This statement should provide the following information, where applicable:

- Accession codes, unique identifiers, or web links for publicly available datasets
- A description of any restrictions on data availability
- For clinical datasets or third party data, please ensure that the statement adheres to our policy

All anonymized behavioral data are available, without restriction at https://osf.io/s92tj/.
Un-thresholded fMRI activation maps have been updated on Neurovault, and are available at https://neurovault.org/collections/MOTXHGZV/

Research involving human participants, their data, or biological material

Policy information about studies with human participants or human data. See also policy information about sex, gender (identity/presentation), and sexual orientation and race, ethnicity and racism.

Reporting on sex and gender	We included participants from both sexes (female/male = 23/12; Age: 22.69±4.44). All personal information were based on self-report.
Reporting on race, ethnicity, or other socially relevant groupings	*Please specify the socially constructed or socially relevant categorization variable(s) used in your manuscript and explain why they were used. Please note that such variables should not be used as proxies for other socially constructed/relevant variables (for example, race or ethnicity should not be used as a proxy for socioeconomic status).*<br>*Provide clear definitions of the relevant terms used, how they were provided (by the participants/respondents, the researchers, or third parties), and the method(s) used to classify people into the different categories (e.g. self-report, census or administrative data, social media data, etc.)*<br>*Please provide details about how you controlled for confounding variables in your analyses.*
Population characteristics	see above
Recruitment	Healthy participants were recruited from the subject pool of the behavioral science lab (https://www.lab.uva.nl/lab) and through poster adverts distributed on the University of Amsterdam (UvA) campus. The sample is therefore representative of young students at UvA, with participants recruited from different departments, ages, sexes. However, it is important to note that the subject pool may not fully represent diverse countries and cultures, which could potentially limit the generalizability of our brain image results.
Ethics oversight	The ethical approval was obtained from the Faculty Ethics Review Board (FMG-UvA) at UvA (reference number: 2018-EXT-9205)

Note that full information on the approval of the study protocol must also be provided in the manuscript.

Field-specific reporting

Please select the one below that is the best fit for your research. If you are not sure, read the appropriate sections before making your selection.

☐ Life sciences ☒ Behavioural & social sciences ☐ Ecological, evolutionary & environmental sciences

For a reference copy of the document with all sections, see nature.com/documents/nr-reporting-summary-flat.pdf

Behavioural & social sciences study design

All studies must disclose on these points even when the disclosure is negative.

Study description	Quantitative experimental design. The present study combined functional magnetic resonance imaging (fMRI) and an optimized reinforcement learning paradigm, in which we systematically manipulated two dimensions of the learning context: the valence of the outcome (gain vs. loss) and the outcome information (partial vs. complete feedback). In the learning task, participants completed three runs of learning task in the MRI scanner. In each run of 80 trials, four fixed pairs of abstract symbols were used to represent four conditions in the two (feedback valence: gain or loss) by two (information: partial or complete) within-subjects design. Participants were asked to choose the one of two options and state their confidence about choosing the symbol that is better on average. At the end of each trial, participants were shown the outcome from the chosen option only in the partial information conditions. Otherwise, both chosen and unchosen outcomes were displayed in the complete information conditions.
Research sample	Healthy participants (n=40, emale/male = 23/12; Age: 22.69±4.44) were recruited from the subject pool of the behavioral science lab (https://www.lab.uva.nl/lab) and through poster adverts distributed on the University of Amsterdam (UvA) campus. The sample is therefore representative of young students at UvA, with participants recruited from different departments, ages, sexes. Sample size was determined according to the behavioral and fMRI studies that used similar task (Palminteri et al., 2015; Lebreton, et al., 2019; Ting et al., 2020).<br><br>References:<br>Palminteri, S., Khamassi, M., Joffily, M., & Coricelli, G. (2015). Contextual modulation of value signals in reward and punishment learning. Nature Communications, 6(1). https://doi.org/10.1038/ncomms9096<br><br>Lebreton, M., Bacily, K., Palminteri, S., & Engelmann, J. B. (2019). Contextual influence on confidence judgments in human reinforcement learning. PLOS Computational Biology, 15(4), e1006973. https://doi.org/10.1371/journal.pcbi.1006973<br><br>Ting, C.-C., Palminteri, S., Engelmann, J. B., & Lebreton, M. (2020). Robust valence-induced biases on motor response and confidence in human reinforcement learning. Cognitive, Affective, & Behavioral Neuroscience. https://doi.org/10.3758/s13415-020-00826-0
Sampling strategy	Participants were randomly sampled from the subject pool of the behavioral science lab (https://www.lab.uva.nl/lab) and through poster adverts distributed on the University of Amsterdam (UvA) campus.
Data collection	Before the experiment, we used a prescreening procedure on Qualtrics and only participants that passed this were invited to come to

Data collection	the MRI scanner and were sent an invitation email and detailed information about the experiment and MRI. In the experiment, both behavioral and imaging data were recorded using Matlab 2015a and 3-T MRI scanner.<br>No one was present during the main experiment, including learning and transfer task. The researcher was not fully blinded to the study hypothesis.
Timing	From October, 2018 to January, 2019
Data exclusions	No behavioral data were excluded. Regarding the neural imaging data, two participants were excluded: one has significant dropout in the functional images and one has invariable responses on confidence rating leading to difficulty on performing followed-up t-contrast.
Non-participation	No participants dropped out or declined participation.
Randomization	All experimental manipulations are within-participants. Therefore, participants were not allocated into experimental groups. Our four key conditions were presented in an interleaved, randomized and unpredictable manner.

Reporting for specific materials, systems and methods

We require information from authors about some types of materials, experimental systems and methods used in many studies. Here, indicate whether each material, system or method listed is relevant to your study. If you are not sure if a list item applies to your research, read the appropriate section before selecting a response.

Materials & experimental systems

n/a	Involved in the study
☒	☐ Antibodies
☒	☐ Eukaryotic cell lines
☒	☐ Palaeontology and archaeology
☒	☐ Animals and other organisms
☒	☐ Clinical data
☒	☐ Dual use research of concern
☒	☐ Plants

Methods

n/a	Involved in the study
☒	☐ ChIP-seq
☒	☐ Flow cytometry
☐	☒ MRI-based neuroimaging

Magnetic resonance imaging

Experimental design

Design type	Event-related design
Design specifications	Each subject completed three runs of 80 trials in a scanning session.<br>The inter-trial interval was between 1000 and 7500ms (mean = 2500ms).
Behavioral performance measures	Choices, reaction times, and confidence judgments were measured.<br>Each measure was normalized (Z-scored) within participant.

Acquisition

Imaging type(s)	Functional and structural images
Field strength	3.0-Tesla Philip Achieva scanner (with 32 channels head array coil).
Sequence & imaging parameters	Structural images: FOV (Field of View): 240x180x220 mm3, Voxel size =1x1x1 mm3, TR = 8.2ms and TE = 3.7ms.<br><br>Functional images: 36 axial echo-planar images (EPI) acquired in ascending sequence with voxel size of 3x3x3 mm3, slice gap = 0.3 mm, TR= 2000ms, TE = 28ms and the flip angle of 76°
Area of acquisition	Whole brain

Diffusion MRI ☐ Used ☒ Not used

Preprocessing

Preprocessing software	spm12
Normalization	Steps: realignment and unwarp, co-registration, segmenting anatomical images, normalization, and smoothing. All functional volumes (from three runs) were realigned to the first volume in the first run and were un-warped with collected field maps. To improve the quality of the following normalization, the mean functional (the output from realignment) and anatomical

images were co-registered. The anatomical image from each subject was segmented into six images (i.e., grey matter, white matter, cerebrospinal fluid, fat tissue and air) using nonlinear deformation fields and SPM12's Tissue Probability Maps (TPMs). All segmented images were then normalized to the Montreal Neurological Institute T1 template (i.e., MNI152) using forward deformation fields from the segmentation output. Finally, the EPI images were normalized and smoothed with a full width half maximum Gaussian kernel of 6-mm (2 times of voxel size of functional images) full-width at half maximum (FWHM) isotropic Gaussian kernel.

Normalization template

NMI152

Noise and artifact removal

To remove motion artifact, all the GLMs also contained six realignment parameters (three directions of translation and three axes of rotation) created during preprocessing stage.

Volume censoring

We did not include any Volume censoring approach to additionally process outlier volumes. All brain images were preprocessed through aforementioned steps (in Normalization section). The noise and artifact were removed by including six realignment parameters (three directions of translation and three axes of rotation) in each GLM.

Statistical modeling & inference

Model type and settings

Mass univariate. Five GLMs modelled separately the four main events composing our prototypical trial: symbol presentation, choice, confidence rating, outcome. These event-related regressors were modeled using boxcar functions with corresponding durations. Across all models, the choice and confidence onsets were respectively modulated with parametric modulators accounting for (1) choice (right or left), (2) the distance between initial and final rating point for rating onset. The five GLMs featured different combinations of symbol presentation structure (single event / condition-specific event) and associated parametric modulators (confidence, Q-vlaues). Across all models, all parametric modulators were z-scored to ensure results from different conditions and regressors were comparable (Lebreton, et al., 2019). Linear contrasts of regression coefficients were designed at the individual level (first-level), and, unless otherwise specified, taken to the group-level random-effect analysis (second-level).

Effect(s) tested

See above

Specify type of analysis: ☐ Whole brain ☐ ROI-based ☒ Both

Anatomical location(s)

Two families of ROIs were created.
A first family of ROIs was built from the significant clusters from the GLM1 confidence activations (VMPFC, dmPFC, Inferior Frontal Gyrus, and Insula); Alternative VMPFC ROIs were also built from independent meta-analyses (Bartra et al., 2013) and from significant clusters from other analyses of the resent study (e.g., voxels significantly correlated to Qc in GLM3).

Statistic type for inference

(See Eklund et al. 2016)

Clusterwise inference with vixel threshold P<0.001 and cluster size >47, achieving PFWE-corrected < 0.05

Correction

FWE

Models & analysis

n/a	Involved in the study
☒	☐ Functional and/or effective connectivity
☒	☐ Graph analysis
☒	☐ Multivariate modeling or predictive analysis
